# The Accuracy and Precision of the Continuously Stored Data from Flash Glucose Monitoring System in Type 2 Diabetes Patients during Standard Meal Tolerance Test

**DOI:** 10.1155/2020/5947680

**Published:** 2020-01-04

**Authors:** Rengna Yan, Huiqin Li, Xiaocen Kong, Xiaofang Zhai, Maoyuan Chen, Yixuan Sun, Lei Ye, Xiaofei Su, Jianhua Ma

**Affiliations:** ^1^Department of Endocrinology, Nanjing First Hospital, Nanjing Medical University, Nanjing 210012, China; ^2^National Heart Research Institute Singapore, National Heart Centre Singapore, Singapore

## Abstract

**Background:**

The purpose of this study was to investigate the accuracy of the continuously stored data from the Abbott FreeStyle Libre flash glucose monitoring (FGM) system in Chinese diabetes patients during standard meal tests when glucose concentrations were rapidly changing. *Subjects and Methods*. Interstitial glucose levels were monitored for 14 days in 26 insulin-treated patients with type 2 diabetes using the FGM system. Standard meal tests were conducted to induce large glucose swings. Venous blood glucose (VBG) was tested at 0, 30, 60, and 120 min after standard meal tests in one middle day of the first and second weeks, respectively. The corresponding sensor glucose values were obtained from interpolating continuously stored data points. Assessment of accuracy was according to recent consensus recommendations with median absolute relative difference (MARD) and Clarke and Parkes error grid analysis (CEG and PEG).

**Results:**

Among 208 paired sensor-reference values, 100% were falling within zones A and B of the Clarke and Parkes error grid analysis. The overall MARD was 10.7% (SD, 7.8%). Weighted least squares regression analysis resulted in high agreement between the FGM sensor glucose and VBG readings. The overall MTT results showed that FGM was lower than actual VBG, with MAD of 22.1 mg/dL (1.2 mmol/L). At VBG rates of change of -1 to 0, 0 to 1, 1 to 2, and 2 to 3 mg/dl/min, MARD results were 11.4% (SD, 8.7%), 9.4% (SD, 6.5%), 9.9% (SD, 7.5%), and 9.5% (SD, 7.7%). At rapidly changing VBG concentrations (>3 mg/dl/min), MARD increased to 19.0%, which was significantly higher than slow changing BG groups.

**Conclusions:**

Continuously stored interstitial glucose measurements with the FGM system were found to be acceptable to evaluate VBG in terms of clinical decision during standard meal tests. The continuously stored data from the FGM system appeared to underestimate venous glucose and performed less well during rapid glucose changes.

## 1. Introduction

Effective glucose monitoring is universally considered as one of the cornerstones of diabetes care. Frequent glucose test is associated with improved glycemic control [1, 2]. Although traditional glucose measurement, self-monitoring of capillary blood glucose (SMBG), has shown a positive impact on metabolic control, the invasiveness, painful fingerpicks, and inconveniences lead to a certain degree of patients' resistance to frequent SMBG [3–6]. The minimally invasive continuous glucose monitoring (CGM) system could record subcutaneous interstitial glucose concentration for 5–7 days long at 5 min intervals, providing not only the information about hypo- and hyperglycemia but also the glucose variability and trends.

Recently, the flash glucose monitoring (FGM) system (FreeStyle FreeStyle® Libre™, Abbott Diabetes Care, Witney, Oxon, UK) provides an alternative to CGM, with the benefits of longer sensor lifetime, lower cost, requiring no user calibration, and providing instantaneous glucose reading. A disposable sensor worn on the back of the upper arm can monitor subcutaneous interstitial glucose concentration for 14 days at 15 minutes intervals. Scanning the sensor wirelessly with a reader can get instant glucose reading. Therefore, FGM makes it more convenient to measure glucose levels. A randomized study, IMPACT, found that patients in the FGM group scanned the sensor at an average of 15.1 times/day, while SMBG testing reduced to a mean of 0.5 times/day. Patients in the control group continued to take SMBG more than 5 times/day [7]. The FGM system FreeStyle Libre is indicated in the European Union to replace blood glucose measurements in many situations.

FGM system is so easy to handle that it can be widely used in prediabetic and diabetic subjects. One crucial issue is the accuracy and precision of this new system. The FGM system can provide two kinds of data. The scanned data are mostly provided by the patients who use the data to adjust their diet, exercise, and even treatment. The continuously stored data reported from clinical software are usually used by researchers to gain glucose profile and assess glycemic control [7–10]. In a clinical trial, 5% of the scanned data showed relative differences of more than ±10% compared with continuously stored data points (median −0.5%) [11]. Such differences might impact the results of studies using this system. However, previous studies analyzing the performance almost used the scanned data [12–14]. Little information is available regarding the accuracy of continuously stored data from the FGM system.

For the SMBG system, the minimum standards for accuracy and reliability of glucose measurement set out in ISO 15197:2013. For the CGM/FGM system, there is no universally accepted protocol to compare performance among subcutaneous interstitial fluid. The Clinical and Laboratory Standards Institute (CLSI) document, Performance Metrics for Continuous Interstitial Glucose Monitoring (POCT05-A), has defined some aspects of CGM testing. The approved guideline, POCT05-A, has pointed to the importance of assessing accuracy not only in steady states but also in two common scenarios: (1) during periods of rapid glucose change and (2) under different glucose concentrations, including extremes of glucose levels [15].

Moreover, some published literature has offered data suggesting that CGM accuracy relative to venous measurements, may need to be adjusted for time lags and may be affected by meal macronutrient composition [13, 14, 16].

Thus in this study, we will analyses the accuracy of the continuously stored data from FGM during the standard meal test when glucose concentrations were changing rapidly.

## 2. Methods

### 2.1. Patients

This was a prospective, single-arm study performed in the outpatient diabetes clinic of Nanjing First Hospital between April and July 2017. The study was approved by the ethics committee and performed in accordance with the Declaration of Helsinki. Written informed consent was obtained before enrollment.

Inclusion criteria were as follows: (1) confirmed T2DM for at least 6 months; (2) age ≥ 18 years; (3) BMI between 18 and 30 kg/m^2^; (4) HbA1c ≤ 9.0% (75 mmol/mol); and (5) had a stable insulin therapy for at least 8 weeks. Exclusion criteria were as follows: (1) patients with a fasting blood glucose level ≤ 3.9 mmol/L or > 11.1 mmol/L; (2) patients who had a history of a major cardiovascular disease event in the previous 6 months; (3) patients with liver dysfunction (aspartate aminotransferase or alanine aminotransferase level of more than two times the upper limit of normal range) or renal dysfunction (creatinine >150 *μ*mol/l or GFR <60 ml/min/1.73 m^2^); (4) patients with severe anemia and hemoglobin disorders (Hb < 60 g/L); and (5) patients who had injection site infection or coagulation disorders. (6) Pregnancy was also excluded.

### 2.2. Study Design

Study participants wore a sensor on the back of the upper arm for up to 14 days. The standard meal test, 75 g instant noodles (305 kcals; consisting 3.3% of fat, 87.9% of carbohydrate, and 8.8% of protein) without oil bag, was conducted in all participants to induce large glucose swings on day 4 and day 9, respectively. Venous blood specimens were collected at 0, 30, 60, and 120 min after standard meal tolerance tests (MTT) for reference tests. The venous blood samples were centrifuged (4000 rpm, 15 min) at 4°C in 5 minutes after collection. Then the serum glucose level was determined by the glucose oxidase method in the central laboratory of Nanjing First Hospital with a Hitachi 7600-120 analyzer (Hitachi Corp, Tokyo, Japan). The instrument was calibrated once a month, and the daily quality control was maintained. The specific time of sample collection was recorded. The continued stored sensor data were reported from clinical software. As the venous blood glucose (VBG) data did not have the same timestamp as a continuously stored data point, the data points recorded immediately before and after the venous blood collection were linearly interpolated. The interpolated value was used to estimate the value of the continuously stored data point if it had been stored at the time of the venous blood collection [11].

### 2.3. Sensor Accuracy Assessments

The continuously stored sensor data and VBG measurements were paired and included for data analysis. Several statistical methods were performed to assess the sensor accuracy of FGM.

Clarke error grid (CEG) [17] and Parkes error grid (PEG) [18] analysis was performed. Reference VBG values were plotted against FGM values within a grid divided into five zones (A, B, C, D, and E), with each zone representing a specific range of clinical significance and risk. The percentage of data points in CEG and PEG zones was calculated [19].

We performed weighted least squares (WLS) regression of FGM regressed against VBG measurements, with weights = VBG^−2^. The square root of the mean square error (MSE) from the regression is an estimate of within-sample coefficient of variation [20]. When the slope of the linear curve was 1 and the intercept was 0, there was no bias between the sensor reading and the VBG value.

Mean absolute relative difference (MARD) is the mean ratio of the absolute difference between the sensor reading and the VBG value. It was performed over the entire glycemic range and for different MTT time points (0, 30, 60, and 120 min) and also for different glucose categories such as 3.9–10 mmol/L (70–180 mg/dL) and >10 mmol/L (>180 mg/dL). MARDs were also calculated in different VBG concentrations rate-of-change categories. Individual absolute relative differences were analyzed and distributed into one of the 5 different rate-of-change categories ranging from −1 mg/dl/min to ≥+3 mg/dl/min in steps of 1 mg/dl/min. The rate-of-change ranges were as follows: >−0.06 to 0 mmol/L/min (>−1 to 0 mg/dL/min), >0 to 0.06 mmol/L/min (>0 to 1 mg/dL/min), >0.06 to 0.11 mmol/L/min (>1 to 2 mg/dL/min), >0.11 to 0.17 mmol/L/min (>2 to 3 mg/dL/min), and >0.17 mmol/L/min (>3 mg/dL/min). Rates of change were calculated based on the VBG concentrations:

(1) $$\begin{array}{c} \text{rate}=\frac{{\text{Glc}}_{i}-{\text{GLc}}_{i-1}}{t_{i}-t_{i-1}}, \end{array}$$

where *t*_*i*_ and *t*_*i*−1_ are the timestamps of the *i*-th and (*i* − 1)-th VBG concentrations, respectively, and Glc_*i*_ and Glc_*i*−1_ are the VBG concentrations corresponding to these VBG timestamps [21].

### 2.4. Statistical Analysis

SPSS 16.0 was used for other statistical analysis. Univariate analyses were performed for descriptive statistics. The *t*-test was used to compare MARDs between one middle day of week 1 and one middle day of week 2 and between euglycemic (3.9–10 mmol/L) and hyperglycemic (>10 mmol/L) groups. MARDs were compared with one-way analysis of variance (ANOVA) at different MTT times (0, 30, 60, and 120 min) and different rate-of-change groups. Paired *t*-test was used to compare areas under the curve between FGM and VBG. The level of significance accepted was 0.05 using two-tailed tests. Clarke error grid and Parkes error grid analysis was performed using R3.6.1 software. The EGA package was mainly used, an algorithm based on the Clark and Parkes error grid principles [17, 18, 22].

## 3. Results

### 3.1. Characteristics of the Study Population

A total of 26 patients (age 59.2 ± 8.4 years old; 8 female and 18 male) with T2DM were enrolled in the study between April and July 2017. Their diabetes duration was 11.2 ± 4.5 years, with BMI 25.7 ± 2.7 kg/m^2^ and HbA1c 7.5 ± 0.9% (58 ± 14 mmol/mol).

### 3.2. Clarke and Parkes Error Grid Analysis

**Figure 1:**
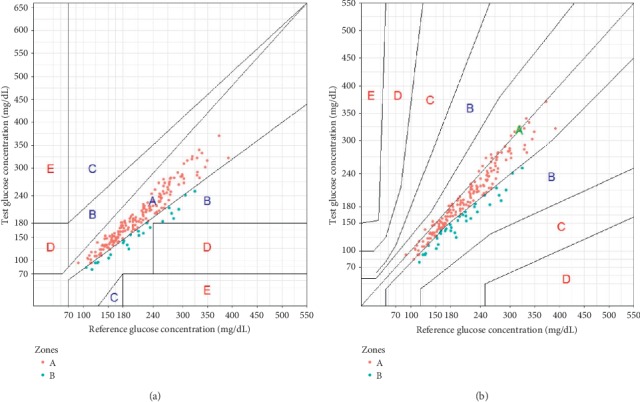
(a) Clarke and (b) Parkes error grid analysis of all the paired data.

Clarke and Parkes error grid analysis was conducted in 208 paired values. The percentage of results in zone A of the Clarke and Parkes error grids were 88.5% and 82.2%, respectively, as shown in Figure 1. For combined zones A and B of the Clarke and Parkes error grids, the percentages were 100%.

### 3.3. Weighted Least Squares Regression Analysis

Regression analysis resulted in high agreement between the FGM sensor glucose compared to VBG readings, with a slope of 0.923 (95% confidence interval (CI) 0.883–0.963), an intercept of −0.268 mmol/L, and a correlation coefficient of 0.954.

### 3.4. MTT Result

**Figure 2:**
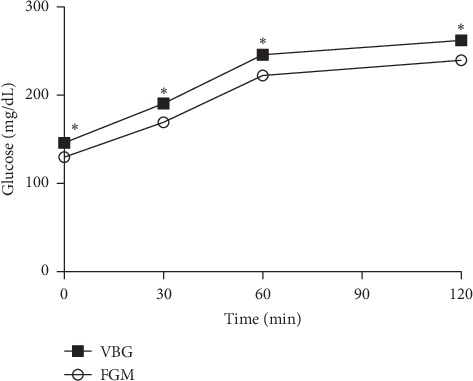
Overall results of MTTs.

The glycemic response measured by both methods is shown in Figure 2. The average VBG and FGM glucose readings were significantly different at any time point (all *p* < 0.001). VBG was significantly higher than FGM at 0, 30, 60, and 120 minutes after glucose loading (146.2 vs. 129.9 mg/dL, 190.6 vs. 169.3 mg/dL, 245.9 vs. 222.3 mg/dL, and 262.1 vs. 239.6 mg/dL, respectively; *p* < 0.001). The mean absolute deviation (MAD) between FGM and VBG was 22.1 mg/dL (1.2 mmol/L). The area under the curve of VBG was also significantly larger than that of FGM (26840 vs. 24220 min·mg/dL; *p* < 0.001).

### 3.5. MARD

As shown in Table 1, the overall MARD was 10.7% (SD, 7.8%). The percentages of MARD ≤20, ≤15, and ≤25 were 88.5%, 73.6%, and 93.3%. For euglycemic (3.9–10 mmol/L) and hyperglycemic (>10 mmol/L) ranges, MARDs were 11.2 (SD, 7.5%) and 10.4 (SD, 8.0%), respectively (*p*=0.495). At different MTT times (0, 30, 60, and 120 min), the MARDs were 12.0 (SD, 8.2%), 11.2 (SD, 8.2%), 10.2 (SD, 7.5%), and 9.5 (SD, 7.2%), respectively (*p*=0.355). MARD also showed no difference between one middle day of week 1 and one middle day of week 2.

**Figure 3:**
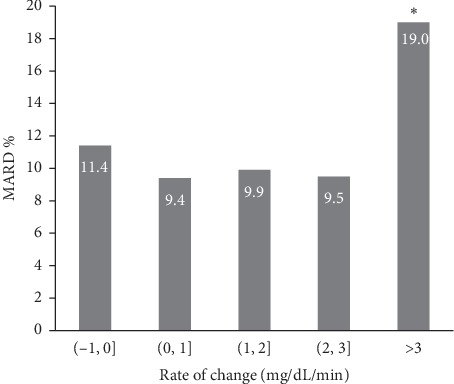
MARD in different VBG rate-of-change categories.

The FGM system showed a rate-of-change dependence to a certain degree. At the lower rate of change groups (−1 to 0, 0 to 1, 1 to 2, and 2 to 3 mg/dl/min), MARD was 11.4% (SD, 8.7%), 9.4% (SD, 6.5%), 9.9% (SD, 7.5%), and 9.5% (SD, 7.7%), respectively. When the rate-of-change rose up to above 3 mg/dl/min, MARD was 19.0% (SD, 10.0%), which was significantly higher than the lower rate of change groups (Figure 3).

## 4. Discussion

This study evaluated the accuracy of the continuously stored data provided by the FreeStyle Libre FGM system during the standard meal tolerance test. The FGM system could provide two kinds of data: continuously stored data and immediately scanned data. The immediately scanned data are useful for patients with diabetes, while continuously stored data are widely used to retrospectively analyze the blood glucose profile by researchers or doctors. Over the past years, several studies have assessed the accuracy of immediately scanned data [14, 23, 24], but only a few about the continuously stored data. This paper was the first to address the continuously stored data as we know.

Among the 208 paired FGM-VBG measurements, 88.5% and 82.2% located in zone A in Clark and Parkes error grid analysis. The percentages in zones A + B were both 100% calculated by the two methods. The 2013 version of ISO 15197 requires ≥95% of measured glucose values to be within zones A or B on the Parkes error grid. The values that fall within zones A and B are clinically acceptable. So, almost all the continuously stored data could be used to evaluate VBG in terms of clinical decision.

The overall MARD was 10.7 (SD, 7.8%), with 10.5 (SD, 7.3%) in week 1 and 11.0 (SD, 8.3%) in week 2. MARD showed no significant difference in glucose concentrations. A lower MARD is seen as representing better sensor performance. There is no universally accepted standardized assessment for ISF glucose sensors. However, the minimum standard for accuracy and reliability of SMBG measurement was set to within ±15% of the comparison measurements at glucose concentration ≥100 mg/dl (5.6 mmol/L). Here, the overall MARD of the FGM system met the minimum standard. We also found that 26.4% and 11.5% of FGM continuously stored data deviated more than 15% and 20%, respectively.

In addition to the glucose concentration, the FGM system also showed a rate-of-change dependence to a certain degree. MARD was 19.0% (SD, 10.0%) at the rate above 3 mg/dl/min, which was significantly higher than the lower rate-of-change groups. When the FGM system measures the glucose concentration in the interstitial tissue, the inaccuracy of rapidly changing VBG concentration is partly due to a physiological time delay. The inaccuracy at rapidly changing VBG concentrations was not associated with the MTT time points. The System's sensor did not show any significant difference in accuracy outcomes relative to the MTT time points (0, 30, 60, and 120 min).

Weighted least squares regression analysis resulted in a high agreement between the FGM sensor glucose and VBG readings. However, the overall MTT results showed that FGM was lower than actual VBG , with MAD of 22.1 mg/dL (1.2 mmol/L). This difference between venous and sensor glucose may be caused by a time delay for physiological reason, especially in the MTT process when the glucose changed rapidly. On the contrary, Sekido et al. [25] reported that FGM was significantly higher than plasma glucose at 30, 60, and 90 minutes after glucose loading. The authors used the immediately scanned data of the FGM system. Another study found that FGM appeared to underestimate plasma glucose in those with overweight/obesity [26]. So, what caused the difference between the immediately scanned data and the continuously stored data of FGM? This problem may need further research.

Due to the practical limitation of obtaining blood, the limitation of the current study is limited venous reference data range (from 5.0 to 21.8 mmol/L). Further research is needed to evaluate the accuracy at low glucose concentrations.

In conclusion, the data presented here indicated that continuously stored interstitial glucose measurements with the FGM system were found to be used to evaluate VBG in terms of clinical decision during standard meal tests. The continuously stored data from the FGM system appeared to underestimate venous glucose and performed less well during rapid glucose changes.

## Figures and Tables

**Table 1:** MARD in different categories.

	MARD (%)	MARD ≤ 30	MARD ≤ 25	MARD ≤ 20	MARD ≤ 15	MARD ≤ 10
Total (*n* = 208)	10.7 ± 7.8	98.1%	93.3%	88.5%	73.6%	53.4%
Week 1 vs week 2						
Week 1 (*n* = 104)	10.5 ± 7.3	99.0%	94.2%	90.4%	76.9%	52.9%
Week 2 (*n* = 104)	11.0 ± 8.3	97.1%	92.3%	86.5%	70.2%	53.8%
*p*	0.661	0.313	0.580	0.385	0.271	0.889
VBG levels (mmol/L)						
3.9–10 (*n* = 76)	11.2 ± 7.5	97.4%	93.4%	90.8%	73.7%	50.0%
>10 (*n* = 132)	10.4 ± 8.0	98.5%	93.2%	87.1%	73.5%	55.3%
*p*	0.495	0.270	0.947	0.425	0.975	0.460
VBG times						
BG 0′ (*n* = 52)	12.0 ± 8.2	96.2%	92.3%	86.5%	67.3%	44.2%
BG 30′ (*n* = 52)	11.2 ± 8.2	98.1%	90.4%	86.5%	73.1%	55.8%
BG 60′ (*n* = 52)	10.2 ± 7.5	98.1%	94.2%	88.5%	78.8%	57.7%
BG 120′ (*n* = 52)	9.5 ± 7.2	100.0%	96.2%	92.3%	75.0%	55.8%
*p*	0.355	0.903	0.792	0.770	0.603	0.498

## Data Availability

The data used to support the findings of this study are available from the corresponding author upon request.
